# Proposed Definitions for Epidemiologic and Clinical Studies of *Mycobacterium avium* Complex Pulmonary Disease

**DOI:** 10.1371/journal.pone.0077385

**Published:** 2013-11-12

**Authors:** Rachel N. Plotinsky, Elizabeth A. Talbot, C. Fordham von Reyn

**Affiliations:** 1 Dartmouth–Hitchcock Medical Center, Lebanon, New Hampshire, United States of America; 2 Providence St. Vincent Medical Center, Portland, Oregon, United States of America; National Institute of Allergy and Infectious Diseases, United States of America

## Abstract

**Background:**

Epidemiologic and clinical studies of *Mycobacterium avium* complex (MAC) pulmonary disease typically use strict ATS/IDSA definitions designed for decisions about treatment. Studies based on these criteria may exclude a substantial number of patients with true disease. We reviewed patients treated for MAC pulmonary disease at an academic medical center to propose revised definitions encompass the full spectrum of MAC pulmonary disease.

**Methods:**

We conducted a retrospective review of patients with MAC pulmonary disease treated from 1993–2006 by pulmonary or infectious disease specialists to assess whether treated patients met current ATS/IDSA microbiologic criteria and dichotomous radiologic classification as nodular/bronchiectatic (NB) or fibrocavitary (FC) disease. We propose a revised set of definitions that include categories of both probable and definite disease to include all treated patients. We further classify patients into dichotomous clinical categories as: “primary MAC” (without antecedent lung disease) or “secondary MAC” (smoking history or antecedent lung disease).

**Results:**

Among 72 treated patients, 74% were female. Median age at diagnosis was 64 years; 41(57%) met ATS/IDSA criteria and 31 (43%) did not, most often for lack of multiple positive cultures. Dichotomous radiologic criteria were met by 48 (67%) patients (36 NB, 12 FC); the remaining 24 (33%) had both NB and FC findings or other abnormalities. Nineteen (26%) were classified as primary and 53 (74%) as secondary MAC (21 COPD, 4 bronchiectasis, 44 smoking history).

**Conclusions:**

We propose revised definitions for epidemiologic and clinical studies of MAC pulmonary disease that describe the full spectrum of disease.

## Introduction

Epidemiologic, clinical and treatment studies of pulmonary disease due to *Mycobacterium avium* complex (MAC) typically rely on diagnostic criteria and treatment recommendations developed and subsequently revised by the American Thoracic Society (ATS) and the Infectious Disease Society of America (IDSA) and intended primarily to inform treatment decisions [1] (Table 1). The definitions require microbiologic confirmation of infection and divide pulmonary disease into dichotomous categories based on radiologic criteria: fibrocavitary (FC) and nodular/bronchiectatic (NB).

**Table 1:** ATS/IDSA criteria for the diagnosis of non-tuberculous mycobacterial lung disease [1].

Clinical (both 1 and 2 required):
(1) Pulmonary symptoms, nodular or cavitary opacities on CXR or high-resolution CT that shows multifocal bronchiectasis with multiple small nodules
And
(2) Appropriate exclusion of other diagnoses
Microbiologic (either 1,2, or 3):
(1) Positive culture from ≥ separate expectorated sputum samples
Or
(2) Positive culture from ≥ bronchial wash or lavage
Or
(3) Biopsy with mycobacterial histopathologic features and positive culture for NTM or biopsy showing mycobacterial histopathologic features and ≥1 sputum or bronchial washings that are culture positive for NTM

Many published studies of pulmonary MAC exclude a substantial number of patients who do not meet full ATS/IDSA criteria [2]. Our experience at an academic medical center suggested that a significant proportion of patients determined by infectious disease or pulmonary specialists to require treatment for MAC pulmonary disease did not meet ATS/IDSA microbiologic criteria. Further, contemporary imaging studies often disclosed features of both FC and NB disease, or other manifestations, making dichotomous radiologic categorization difficult.

In the present report, we summarize our 13-year experience in the diagnosis and treatment of MAC pulmonary disease at an academic medical center. We report on all treated patients, including those who did not meet ATS/IDSA definitions. Based on our experience we propose that a larger prospective study be conducted using expanded definitions for epidemiologic and clinical studies with a new dichotomous system for classifying cases based on the presence or absence of underlying lung disease at the time of MAC diagnosis.

## Methods

### Ethics statement

This study was exempted from Institutional Review Board evaluation by the Committee for the Protection of Human Subjects of Dartmouth. This study was exempted from obtaining written consent from patients since the research posed no more than minimal risk to the subjects, the waiver of consent did not adversely affect the rights and welfare of subjects, and the research could not practicably be carried out without the waiver of consent.

### Patients

We performed medical record review of both electronic and paper records for all HIV-negative patients aged >18 years who were diagnosed with *Mycobacterium avium* complex (MAC) pulmonary disease from 1993 through 2006 and were treated by pulmonary (n = 20), infectious disease (n = 42), or both (n = 10) specialists at an academic medical center. We identified patients using the International Statistical Classification of Diseases (ICD-9) code for MAC. Patients were included if they had MAC pulmonary disease and were either referred to the medical center after diagnosis or were diagnosed at the medical center. For each patient, we abstracted demographic, clinical, radiologic, and laboratory data, past and current treatment details, adverse drug reactions, and outcomes data from the medical record. If a patient had both chest CT scan and chest x-rays performed, CT scan results were used preferentially over x-ray results to classify patients. All imaging interpretations were abstracted from the official radiology report.

### Microbiology

Sputum, bronchoalveolar lavage, and tissue samples were decontaminated, inoculated onto Lowenstein-Jensen and Middlebrook MH11 medium, and placed in MB/BacT broth bottles with antibiotic supplement (bioMerieux, Durham, NC). Positive cultures were identified, and differentiated by growth characteristics, conventional biochemical tests, and nucleic acid hybridization for MAC complex (Accuprobe, Gen-Probe, San Diego, CA).

### Definitions

We determined whether cases met American Thoracic Society/Infectious Disease Society of America (ATS/IDSA) diagnostic criteria for MAC pulmonary disease (Table 1) and whether they had findings of fibrocavitary or nodular/bronchiectatic disease by radiography.

We classified MAC pulmonary disease as primary or secondary. Primary disease was defined as disease occurring in persons without known antecedent pulmonary disease at time of MAC diagnosis. Secondary disease was defined as disease occurring in a patient with known antecedent lung disease at time of MAC diagnosis such chronic obstructive pulmonary disease, asthma, or pre-existing bronchiectasis. Patients with known history of smoking were also classified as secondary. Patients with unknown smoking history or non-pulmonary co-morbidities (e.g., Behcet's disease, rheumatoid arthritis) were classified as having primary disease. If patients were noted to have bronchiectasis for the first time at the time of MAC diagnosis, they were classified as primary, whereas if they had a diagnosis of bronchiectasis prior to their MAC diagnosis they were classified as having secondary disease.

### Data analysis

Data were entered into an Excel database and statistical analysis was performed using OpenEpi v2.3.1 shareware (http://www.openepi.com/Menu/OpenEpiMenu.htm) and Epi Info 7 software (CDC, Atlanta, GA). We used chi square testing to compare proportions. We used ANOVA parametric tests for comparison of continuous data. We considered p (2-tail) <0.05 to be statistically significant.

## Results

### Diagnosis and clinical classification

We identified 72 patients diagnosed and treated for MAC pulmonary disease. Forty-one patients (57%) met ATS/IDSA diagnostic criteria for NTM lung disease and 31 (43%) did not meet criteria (Table 2). Patients who failed to meet criteria usually met the symptom criterion, but were missing radiologic (e.g., had infiltrates without BN or FC characteristics) or microbiologic criteria (e.g., had only a single positive expectorated sputum). Fifty-nine patients (82%) underwent computed tomography scanning, 13 (18%) had chest x rays. Radiologic findings are summarized in Table 3.

**Table 2:** Classification of 72 patients with primary and secondary MAC by ATS/IDSA diagnostic criteria.

	All patients	Primary	Secondary	P-value
	(n = 72)	(n = 19)	(n = 53)	
Met ATS/IDSA criteria	41 (57%)	9 (47%)	32 (60%)	
Did not meet ATS/IDSA criteria	31 (43%)	10 (53%)	21 (40%)	0.33
Missing criteria*				
Symptoms	3/31 (4%)	1/10 (10%)	2/21 (10%)	0.97
Microbiology	17/31 (55%)	5/10 (50%)	12/21 (57%)	0.71
Radiology	14/31 (45%)	5/10 (50%)	9/21 (43%)	0.71

* categories not mutually exclusive.

**Table 3:** Radiologic findings in 72 patients with MAC pulmonary disease.

Characteristic	All patients	Primary	Secondary	P-value
	(n = 72)	(n = 19)	(n = 53)	
Fibrocavitary (FC)	12	4	8	0.55
Nodular/Bronchiectatic (NB)	36	9	27	0.79
Other	24	6	18	0.85
Both FC and NB	6/24 (25%)	0 (0%)	6/18 (33%)	0.17
Consolidation or infiltrate	12/14 (50%)	4/6 (67%)	8/18 (44%)	0.60
Mass	3/24 (13%)	0 (0%)	3/18 (17%)	0.33

Nineteen patients (26%) were classified as primary MAC, and 53 patients (74%) as secondary MAC (Table 2). Antecedent lung disease for the 53 secondary MAC patients included COPD, bronchiectasis, and asthma.

### Patients and symptoms

Demographic features and symptoms of patients with primary and secondary MAC are shown in Table 4. The majority of patients (74%) were female. Median age at diagnosis was 64 years (range 38–88). The most common presenting symptom for both groups was cough. With the exception of hemoptysis, there were no statistically significant differences between the two groups.

**Table 4:** Demographic features and symptoms of primary and secondary MAC in 72 patients.

Characteristic	All patients	Primary	Secondary	P-value	Met ATS/IDSA criteria	Did not meet ATS/IDSA criteria	P-value
	(n = 72)	(n = 19)	(n = 53)		(n = 41)	(n = 31)	
Female sex	53 (74%)	16 (84%)	37 (70%)	0.22	31 (76%)	22 (71%)	0.66
Median age (range)		68 (50–83)	63 (38–88)	0.38	63 (42–86)	69 (38–88)	0.36
Prior MAC treatment	11 (15%)	3 (16%)	8 (15%)	0.94	6 (15%)	5 (16%)	0.87
Presenting symptoms*							
Cough	60 (83%)	15 (79%)	45 (85%)	0.55	36 (88%)	24 (77%)	0.24
Hemoptysis	9 (13%)	5 (26%)	4 (8%)	0.03	3 (7%)	6 (19%)	0.12
Chest pain	4 (6%)	0 (0%)	4 (8%)	0.21	2 (5%)	2 (6%)	0.77
Dyspnea	14 (19%)	2 (11%)	12 (16%)	0.25	9 (22%)	5 (16%)	0.54
Fever	3 (4%)	0 (0%)	3 (6%)	0.29	1 (2%)	2 (6%)	0.40
Weight loss	9 (13%)	2 (11%)	7 (13%)	0.76	4 (10%)	5 (16%)	0.42
Night sweats	3 (4%)	0 (0%)	3 (6%)	0.29	2 (5%)	1 (3%)	0.73
Fatigue	8 (11%)	0 (0%)	8(15%)	0.07	4 (10%)	4 (13%)	0.67
None	2 (3%)	1 (5%)	1 (2%)	0.44	0 (0%)	2 (6%)	0.10
Other	1 (1%)	0 (0%)	1 (2%)	0.55	1 (2%)	0 (0%)	0.38

* categories not mutually exclusive.

## Discussion

This review of 13 years' experience with the treatment of MAC pulmonary disease at an academic medical center indicates that a substantial proportion of patients deemed by pulmonary or infectious disease specialists to require treatment do not meet current ATS/IDSA criteria for MAC pulmonary disease. Missing criteria fell into two major categories: microbiologic and radiologic. In many cases only a single expectorated sputum was positive for MAC in a patient with pulmonary symptoms, but treatment was compelled by symptoms, radiologic evidence of a new pulmonary process, and no alternate diagnosis. In some cases only a single sputum sample could be obtained and in other cases only one of multiple specimens was positive. Radiologic manifestations sometimes included infiltrates, consolidation or masses [2]–[5] rather then the NB or FC features specified in the definitions. In a recent study of patients with lung disease due to NTM (90% *M. avium* complex) the radiologic manifestation was infiltrate in 54% of patients and less than 10% had bronchiectasis or a cavity [6].

Clinical guidelines for the diagnosis of diseases without a single gold standard diagnostic typically attempt to combine a constellation of features which are believed by expert opinion to be sufficiently sensitive and specific to warrant treatment, and necessarily include subcategories of definite and probable (e.g. infective endocarditis [7]). Guidelines based on expert opinion must be calibrated to consider the risks of treatment vs no treatment based on the potential severity of the disease in question. For diseases such as MAC pulmonary disease, without high short-term mortality and with significant side effects of treatment, criteria can be designed to have high specificity at the expense of relatively lower sensitivity. Although there is no single gold standard diagnostic for pulmonary MAC, studies suggest that multiple positive sputum samples have high specificity based either on association with disease progression or with an increased probability of characteristic radiologic findings [8], [9]. This is the basis for requiring multiple positive samples if expectorated sputum is the only available sample source to meet the microbiologic criteria, and the basis for requiring radiologic findings that are characteristic of MAC pulmonary involvement.

ATS/IDSA criteria have been developed primarily to guide treatment decisions yet most epidemiologic and clinical studies also rely on these definitions. Importantly the definitions were not based on prospective data, but on expert opinion, including that of one of the present authors. Studies using these definitions find that only 20–50% of MAC isolates are associated with clinical features that meet ATS/IDSA criteria [10]–[13]. The assumption is that patients who do not meet criteria do not have disease, typically because only a single positive MAC isolate is available. However in a comprehensive study from Oregon in which detailed chart reviews were conducted on patients with even a single MAC isolate, patients who did not meet criteria had characteristics that were similar to those who did meet criteria. The authors considered the possibility that these patients had true disease [14]. A study from four integrated health care systems also found similar characteristics among patients with MAC isolates who did or did not meet ATS/IDSA criteria, again raising the possibility of low sensitivity of current definitions [13]. In a recent study of 120 patients from Finland with long term follow-up only 50% of patients with a single positive culture for MAC met ATS/IDSA criteria, and treatment was offered to almost 50% of those who did not meet criteria. Long term prognosis was not different in those who did or did not meet criteria, but was determined principally by underlying disease, supporting our recommendation to make this the basis for categorization of pulmonary MAC [2].

We found that experienced pulmonary and infectious disease specialists at an academic medical center performing comprehensive patient evaluation, including symptoms, imaging studies, microbiology and exclusion of other causes of pulmonary disease frequently diagnosed and treated MAC pulmonary disease that did not meet ATS/IDSA criteria. Although treatment of patients who did not meet criteria has been documented [14] and some studies have included cases based on documented use of antimycobacterial drug regimens [15], we are not aware of studies that have analyzed rates of disease based on a specialist's decision to treat. Our study is retrospective, but if confirmed by a larger study with prospective assessment, it suggests the need to expand and revise the diagnostic criteria for a more accurate profile of pulmonary MAC disease patterns.

Based on our review, we suggest two sets of modifications for epidemiologic and clinical studies. The first is to define “definite MAC pulmonary disease” based on the current criteria and to add a category of “probable MAC pulmonary disease” as shown in Table 5 to allow capture of patients with only a single positive sputum or with radiologic features other than NB or FC. This addition would encourage careful consideration of treatment for patients who do not meet current criteria but have a strong probability of bona fide MAC pulmonary disease. Further, the probable category would allow inclusion of data on a wider spectrum of patients in published reports on the epidemiology, clinical features or treatment of MAC pulmonary disease.

**Table 5:** Proposed diagnostic criteria for definite, probable, or possible pulmonary MAC and clinical classification as primary or secondary pulmonary MAC.

**Definite MAC:** specified findings from all 4 definite categories.
**Probable MAC:** specified findings from all 4 probable categories or 1–3 definite categories and the remainder from probable categorie(s)
**Possible MAC:** single positive sputum and documentation of treatment
**1.Symptoms**
*Definite:* pulmonary symptoms
*Probable:* pulmonary symptoms
**2. Imaging**
*Definite:* nodular or cavitary opacities on CXR or high-resolution CT that shows multifocal bronchiectasis with multiple small nodules
*Probable:* infiltrate, consolidation, or mass on CXR or CT scan
**3. Microbiology**
*Definite:*
(1) Positive culture from ≥2 separate expectorated sputum samples
Or
(2) Positive culture from ≥1 bronchial wash or lavage
Or
(3) Biopsy with mycobacterial histopathologic features and positive culture for NTM or biopsy showing mycobacterial histopathologic features and ≥1 sputum or bronchial washing that is culture positive
*Probable:* positive culture from 1 expectorated sputum sample
**4. Exclusions**
*Definite:* Appropriate exclusion of other possible causes of the clinical complex
*Probable:* Appropriate exclusion of other possible causes of the clinical complex
CLINICAL CLASSIFICATION
1. Primary: no smoking history and no known underlying pulmonary disease prior to the diagnosis of MAC pulmonary disease
2. Secondary: Smoking history or known underlying pulmonary disease prior to the diagnosis of MAC pulmonary disease (e.g. COPD, cystic fibrosis)

Other authors have conducted analyses of cases defined as “possible” [13], [16]. In the study from the United States a single positive sputum was used to define possible; other features were not required, although radiologic features were similar to cases meeting ATS/IDSA definitions [13]. Our recommendation is to define both microbiologic and expanded radiologic criteria for a second category so that it can be considered “probable” pulmonary MAC. A category of possible MAC would include patients with a single isolate who were treated regardless of whether symptoms or radiologic criteria were present or noted.

The second recommendation would be to change from a dichotomous classification based on radiologic findings to a dichotomous clinical classification based on the presence or absence of recognized underlying lung disease. There is no firm basis for assuming that when the same mycobacterial species causes different imaging patterns that this results in clinically meaningful separation of patient groups. For example both primary disease and reactivation with *M. tuberculosis* may cause cavitation [17]. Numerous studies have suggested that non-smoking women over age 50 have a different form of disease than the predominantly male patients with MAC pulmonary disease superimposed on underlying COPD, and may have a distinct pathophysiology [18], [19]. Women with this disease pattern may have cavities or nodules [19]. Further, as our findings indicate, patients may have both FC and NB findings preventing dichotomous classification, or may have other radiologic patterns associated with MAC pulmonary disease. Radiologic imaging remains important since it may also identify underlying lung disease to classify patients as secondary MAC.

In contrast, most patients could be easily classified as having primary or secondary disease, and these categories are more likely to facilitate better understanding of pathophysiology. For example, if acid reflux is the proximate cause of MAC pulmonary infection in non-smoking patients over age 50 [20], or if MAC is associated with a particular morphologic phenotype [21], [22], we believe these associations are more likely to be elucidated by studies of all subjects with apparent primary disease than by studies on the subset subjects with a particular radiologic pattern. Primary and secondary disease patients are also likely to have different risks of adverse outcome based on pre-existing pulmonary disease [23].

One challenge in the proposed clinical classification is whether patients who have bronchiectasis first identified along with a new diagnosis of MAC pulmonary disease had this pulmonary pathology as a risk factor for MAC or as a consequence of MAC. Clearly mycobacterial infection itself can result in bronchiectasis as is the case with tuberculosis [24], [25]. However, bronchiectasis is also known to facilitate pulmonary infection [26], and may be a form of predisposing lung disease for MAC. We segregated patients based on whether bronchiectasis had been diagnosed before MAC, but patients may not have had MAC cultures obtained until bronchiectasis had been present for some time. Thus, some misclassification is likely.

Our study has several limitations. The analysis of whether patients met ATS/IDSA criteria is based on data, but this clinical data was collected retrospectively and is therefore subject to the limitations of this approach (e.g., data about symptoms such as weight loss or risk factors such as smoking may have not been recorded in the notes). Not all patients had high resolution CT scans which might have detected additional instances of FC or NB disease patterns. In the absence of a long term prospective study starting with healthy subjects, it is impossible to determine if bronchiectasis first detected at the time of MAC diagnosis was a risk factor for or consequence of MAC disease. Further, we did not have data on the number of sputum samples collected for each patient, and we did not review patients with positive sputum cultures who were judged not to require treatment. Our sample size was too small to report or analyze differences in outcomes between treated patients in the two categories.

Our proposal for revised definitions is not based on prospective data, but rather on our experience with the limitations of the ATS/IDSA criteria, literature review, and inference from development of definitions for other diseases without a single gold standard diagnostic. Importantly, the proposed definitions are not intended to serve as a guideline for treatment but rather as a more comprehensive categorization of patients for use in clinical and epidemiologic studies. Treatment decisions are best made by specialists who can use definitions as one part of an expert evaluation that includes an assessment of the severity and pace of a newly identified pulmonary process along with exclusion of other potential causes of lung disease.

In summary, a substantial proportion of patients diagnosed and treated for MAC pulmonary disease by pulmonary or infectious disease specialists at an academic medical center do not meet current ATS/IDSA criteria for MAC pulmonary disease and cannot easily be classified based on radiologic criteria. Our findings suggest a need to study the addition of criteria for “probable” MAC and to shift to a clinical classification of primary or secondary MAC based on the presence or absence of recognized underlying lung disease. The use of expanded and revised definitions in prospective studies may provide an improved understanding of the full epidemiologic and clinical spectrum of pulmonary MAC disease.

## References

[1] Griffith DE, Aksamit T, Brown-Elliott B, Catanzaro A, Daley C, et al (2007) An Official ATS/IDSA Statement: Diagnosis, Treatment, and Prevention of Nontuberculous Mycobacterial Diseases. Am J Respir Crit Care Med 175: 367–416. 17277290 10.1164/rccm.200604-571ST

[2] Lim J, Lyu J, Choi CM, Oh YM, Lee SD, et al (2010) Non-tuberculous mycobacterial diseases presenting as solitary pulmonary nodules. Int J Tuberc Lung Dis 14: 1635–40. 21144251

[3] Embil J, Warren P, Yakrus M, Stark R, Corne S, et al (1997) Pulmonary illness associated with exposure to *Mycobacterium-avium* complex in hot tub water: Hypersensitivity pneumonitis or infection? Chest 111: 813–16. 9118726 10.1378/chest.111.3.813

[4] Hanak V, Kalra S, Aksamit TR, Hartman TE, Tazelaar HD, et al (2006) Hot tub lung: presenting features and clinical course of 21 patients. Respir Med 100: 610–5. 16194601 10.1016/j.rmed.2005.08.005

[5] Moore EH (1993) Atypical mycobacterial infection in the lung: CT appearance. Radiology 187: 777–82. 8497629 10.1148/radiology.187.3.8497629

[6] Kendall BA, Varley CD, Choi D, Cassidy PM, Hedberg K, et al (2011) Distinguishing tuberculosis from nontuberculous mycobacteria lung disease, Oregon, USA. Emerg Infect Dis 17: 506–9. 21392445 10.3201/eid1703.101164 PMC3166013

[7] von Reyn CF, Levy BS, Arbeit RD, Friedland G, Crumpacker CS (1981) Infective endocarditis: an analysis based on strict case definitions. Ann Intern Med 94: 505–18. 7011141 10.7326/0003-4819-94-4-505

[8] Tsukamura M (1991) Diagnosis of disease caused by Mycobacterium avium complex. Chest 99 (3) 667–9. 1995223 10.1378/chest.99.3.667

[9] Olivier KN, Weber DJ, Lee JH, Handler A, Tudor G, et al (2003) Nontuberculous mycobacteria. II: nested-cohort study of impact on cystic fibrosis lung disease. Am J Respir Crit Care Med 167: 835–40. 12433669 10.1164/rccm.200207-679OC

[10] Daley CL, Griffith DE (2010) Pulmonary non-tuberculous mycobacterial infections. Int J Tuberc Lung Dis 14: 665–71. 20487602

[11] Thomson RM (2010) Changing epidemiology of pulmonary nontuberculous mycobacteria infections. Emerg Infect Dis 16: 1576–83. 20875283 10.3201/eid1610.091201 PMC3294381

[12] Cassidy PM, Hedberg K, Saulson A, McNelly E, Winthrop KL (2009) Nontuberculous mycobacterial disease prevalence and risk factors: a changing epidemiology. Clin Infect Dis 49: e124–9. 19911942 10.1086/648443

[13] Prevots DR, Shaw PA, Strickland D, Jackson LA, Raebel MA, et al (2010) Nontuberculous mycobacterial lung disease prevalence at four integrated health care delivery systems. Am J Respir Crit Care Med 182: 970–6. 20538958 10.1164/rccm.201002-0310OC PMC2970866

[14] Winthrop KL, McNelley E, Kendall B, Marshall-Olson A, Morris C, et al (2010) Pulmonary nontuberculous mycobacterial disease prevalence and clinical features: an emerging public health disease. Am J Respir Crit Care Med 182: 977–82. 20508209 10.1164/rccm.201003-0503OC

[15] Hernandez-Garduno E, Rodrigues M, Elwood RK (2009) The incidence of pulmonary non-tuberculous mycobacteria in British Columbia, Canada. Int J Tuberc Lung Dis 13: 1086–93. 19723396

[16] Andrejak C, Thomsen VO, Johansen IS, Riis A, Benfield TL, et al (2010) Nontuberculous pulmonary mycobacteriosis in Denmark: incidence and prognostic factors. Am J Respir Crit Care Med 181: 514–21. 20007929 10.1164/rccm.200905-0778OC

[17] Geng E, Kreiswirth B, Burzynski J, Schluger NW (2005) Clinical and radiographic correlates of primary and reactivation tuberculosis: a molecular epidemiology study. JAMA 293: 2740–5. 15941803 10.1001/jama.293.22.2740

[18] Reich JM, Johnson RE (1992) Mycobacterium avium complex pulmonary disease presenting as an isolated lingular or middle lobe pattern. The Lady Windermere syndrome 101: 1605–9. 10.1378/chest.101.6.1605 1600780

[19] Prince DS, Peterson DD, Steiner RM, Gottleib JE, Scott R, et al (1989) Infection with *Mycobacterium avium* complex in patients without predisposing conditions. New Engl J Med 321: 863–8. 2770822 10.1056/NEJM198909283211304

[20] Thomson RM, Armstrong JG, Looke DF (2007) Gastroesophageal reflux disease, acid suppression, and Mycobacterium avium complex pulmonary disease. Chest 131: 1166–72. 17426224 10.1378/chest.06-1906

[21] Kim RD, Greenberg DE, Ehrmantraut ME, Holland SM (2008) Pulmonary nontuberculous mycobacterial disease. Am J Respir Crit Care Med 178: 1066–74. 18703788 10.1164/rccm.200805-686OC PMC2720143

[22] Iseman MD, Buschman DL, Ackerson LM (1991) Pectus excavatum and scoliosis: thoracic abnormalities associated with pulmonary disease caused by *Mycobacterium avium* complex. Am Rev Respir Dis 144: 914–6. 1928970 10.1164/ajrccm/144.4.914

[23] Aksamit TR (2002) Mycobacterium avium complex pulmonary disease in patients with pre-existing lung disease. Clin Chest Med 23: 643–53. 12371000 10.1016/s0272-5231(02)00022-9

[24] Shin MS, Ho KJ (1989) Computed tomography of bronchiectasis in association with tuberculosis. Clin Imaging 13: 36–43. 2743190 10.1016/0899-7071(89)90123-x

[25] Jordan TS, Spencer EM, Davies P (2010) Tuberculosis, bronchiectasis and chronic airflow obstruction. Respirology 15: 623–8. 20409028 10.1111/j.1440-1843.2010.01749.x

[26] Prasad M, Tino G (2007) Bronchiectasis, part 1: Presentation and diagnosis. J Respir Dis 28: 545–55.

